# Fried phenotype of frailty: cross-sectional comparison of three frailty stages on various health domains

**DOI:** 10.1186/s12877-015-0078-0

**Published:** 2015-07-09

**Authors:** Linda P. M. Op het Veld, Erik van Rossum, Gertrudis I. J. M. Kempen, Henrica C. W. de Vet, KlaasJan Hajema, Anna J. H. M. Beurskens

**Affiliations:** Centre of Research Autonomy and Participation for Persons with a Chronic Illness, Faculty of Health, Zuyd University of Applied Sciences, Nieuw Eyckholt 300, 6419DJ Heerlen, The Netherlands; Department of Health Services Research, CAPHRI, School for Public Health and Primary Care, Maastricht University, Duboisdomein 30, 6229GT Maastricht, The Netherlands; Department of Epidemiology and Biostatistics, EMGO Institute for Health and Care Research, VU University Medical Center, Amsterdam, The Netherlands; Community Health Service South Limburg, Academic Collaborative Centres Public Health (ACC), Geleenbeeklaan 2, 6166 GR Sittard-Geleen, The Netherlands; Department of Family Practice, CAPHRI, School for Public Health and Primary Care, Maastricht University, Peter Debeyeplein 1, 6229 HA Maastricht, The Netherlands

**Keywords:** Frailty, Frailty phenotype, Frailty stages, Functional abilities of older persons

## Abstract

**Background:**

The population ageing in most Western countries leads to a larger number of frail older people. These frail people are at an increased risk of negative health outcomes, such as functional decline, falls, institutionalisation and mortality. Many approaches are available for identifying frailty among older people. Researchers most often use Fried and colleagues’ description of the frailty phenotype. The authors describe five physical criteria. Other researchers prefer a combination of measurements in the social, psychological and/or physical domains. The aim of this study is to describe the levels of social, psychological and physical functioning according to Fried’s frailty stages using a large cohort of Dutch community-dwelling older people.

**Methods:**

There were 8,684 community-dwelling older people (65+) who participated in this cross-sectional study. Based on the five Fried frailty criteria (weight loss, exhaustion, low physical activity, slowness, weakness), the participants were divided into three stages: non-frail (score 0), pre-frail (score 1–2) and frail (score 3–5). These stages were related to scores in the social (social network type, informal care use, loneliness), psychological (psychological distress, mastery, self-management) and physical (chronic diseases, GARS IADL-disability, OECD disability) domains.

**Results:**

63.2 % of the participants was non-frail, 28.1 % pre-frail and 8.7 % frail. When comparing the three stages of frailty, frail people appeared to be older, were more likely to be female, were more often unmarried or living alone, and had a lower level of education compared to their pre-frail and non-frail counterparts. The difference between the scores in the social, psychological and physical domains were statistically significant between the three frailty stages. The most preferable scores came from the non-frail group, and least preferable scores were from the frail group. For example use of informal care: non-frail 3.9 %, pre-frail 23.8 %, frail 60.6 %, and GARS IADL-disability mean scores: non-frail 9.2, pre-frail 13.0, frail 19.7.

**Conclusion:**

When older people were categorised according to the three frailty stages, as described by Fried and colleagues, there were statistically significant differences in the level of social, psychological and physical functioning between the non-frail, pre-frail and frail persons. Non-frail participants had consistently more preferable scores compared to the frail participants. This indicated that the Fried frailty criteria could help healthcare professionals identify and treat frail older people in an efficient way, and provide indications for problems in other domains.

## Background

An ageing society is a common phenomenon. The increasing proportion of older people in most Western countries leads to a larger number of people who are old and frail. These frail older people are at an increased risk of negative health outcomes, such as functional decline, falls, institutionalisation and mortality [1]. Over the last decade, the interest in frailty has grown [2]. The main reason is the belief that early identification of those at risk could help to delay or prevent the adverse outcomes of frailty. Despite considerable research on frailty, there is still debate on the nature, definition, prevalence, and the characteristics of older people in various frailty ‘stages’ [1, 3].

Three main approaches to conceptualise frailty have been distinguished. One approach considers frailty to be a decline in physical functioning. The frailty phenotype, as described by Fried and colleagues, is based on five pre-defined physical frailty criteria, which are well known and most frequently used by researchers [4, 5]. Another approach is to look at frailty as the accumulation of deficits across various domains (e.g. cognition, physical functioning, self-rated health, smoking history, and laboratory results). The Frailty Index, developed by Rockwood and colleagues, is often used for this approach and it is characterised by the use of a non-fixed set of clinical conditions and diseases [6, 7]. A third approach also assumes that multiple domains (social, psychological, physical) are involved in the concept of frailty, with researchers using a pre-defined set of questions related to each domain (e.g. Tilburg Frailty Indicator, Groningen Frailty Indicator) [8, 9].

Each approach has its advantages and disadvantages. In the present study, we are looking for a brief and simple tool (i.e. a self-report questionnaire with a limited number of items) that is feasible for use in large populations of community-dwelling older people. The Fried frailty criteria seem to reflect such a tool. Although the Fried criteria were originally not developed as a self-report questionnaire, researchers nowadays often use (partly) modified questionnaires that are based on the frailty phenotype (e.g. Barreto and colleagues, Macklai and colleagues) [10, 11].

The five frailty criteria are weight loss, exhaustion, low physical activity, slowness and weakness. The sum score of these five criteria classifies people into one of three frailty stages (or groups): not frail (score 0), pre-frail (score 1–2) and frail (score 3–5). Fried and colleagues described the characteristics of these three groups using a cohort of United States citizens. The trend was that frail people were older, more likely to be female, suffered from more diseases (except cancer), reported higher rates of disability, were less educated, had lower income, were in poorer health, had more cognitive impairments and experienced higher levels of depressive symptoms compared to their pre-frail and non-frail counterparts [5]. Results from the pre-frail people were intermediate, falling between the scores of the frail and non-frail people (except for cancer). In addition, outcomes of the Survey of Health, Aging and Retirement in Europe (SHARE), which also used the Fried criteria to assess frailty in populations from 11 European countries, showed that frail people were more likely to be female and report more disability problems compared to their pre-frail and non-frail counterparts [11]. The particular characteristics of interest in both aforementioned studies were demographics, and aspects in the physical domain, as well as (chronic) diseases. Studies from Bandeen-Roche and colleagues [12], Ble and colleagues [13] and Cawthon and colleagues [14] also used the five Fried frailty criteria to differentiate between groups, focusing on similar characteristics of interest. It is still unclear whether this limited scope is sufficient for identifying different profiles of functioning of frail, pre-frail and non-frail older people. Levels of social and psychological functioning might also, for example, play an important role in the development of frailty.

Additional knowledge regarding whether such social and psychological factors could add to the discriminative power of the three Fried frailty stages will be very useful for both healthcare professionals and researchers. Up until now, the psychological and social factors relative to the frailty stages have not been extensively studied. If these stages also show variations in these domains, this could help healthcare professionals in efficiently identifying and treating frail older people. If a patient is (pre-)frail according to the Fried criteria, it could alert them to the existence of problems in other domains as well. Moreover, as the number of items of the Fried frailty criteria is limited, the use of this short instrument is much more efficient than many other frailty measures.

The aim of this study is to describe the levels of social, psychological and physical functioning according to the three Fried frailty stages using a large cohort of Dutch community-dwelling older people. We also studied possible gender differences in these levels of functioning.

## Methods

A cross-sectional study was conducted among community-dwelling older people in Limburg, a province in the southern part of The Netherlands. The medical ethical committee Atrium-Orbis-Zuyd approved this study (12-N-129). Selection of the study population was made from the Health Monitor, an extensive postal general health questionnaire which is sent every four years by the Community Health Service to a large sample of community-dwelling people in the Netherlands [15].

### Study population

For the measurement using the Health Monitor in Limburg, during the fall of 2012, 56,000 people aged 55 years and over were selected. Selection was random for all age groups, except for those over 75 years. This population was overrepresented in the sample in order to obtain sufficient data among the oldest age group living at home. People living in neighbourhoods with a low socioeconomic status were overrepresented as well. Respondents were asked to give their consent for using their data for our study.

The response rate for the Health Monitor was 54 % (n = 30,130). Of the respondents, 13,521 gave permission for the use of their data in our study. The selection was also restricted to those who were 65 years and older, because this is the age group in which the Fried criteria were originally developed [5]. After excluding the questionnaires that were filled out by a person other than the addressee and those questionnaires with a significant amount of missing data, a total of 8,684 people participated in our study.

### Measurements

The Health Monitor is comprised of a broad range of questions. In addition to demographic characteristics (age, gender, marital status and level of education), questions included the Fried frailty criteria, (chronic) diseases, use of healthcare services, use of informal care and items about social, psychological and physical functioning.

#### Fried frailty criteria

Fried and colleagues developed five criteria (weight loss, exhaustion, low physical activity, slowness and weakness) to be used for identifying frail older people [5]. In contrast with the original criteria, we replaced the two physical measurements of slowness and weakness by questions. *Weight loss* was measured using the question: “In the last year, have you lost more than 4.5 kilograms unintentionally? (i.e. not due to dieting or exercise)”. This question is the same as proposed by Fried and colleagues, only pounds were replaced by kilograms. This criterion was met when the participant answered “yes”. *Exhaustion* was measured using two questions from the Center for Epidemiologic Studies Depression (CES-D) scale: “How often did you feel that everything you did was an effort?” and “How often did you feel that you could not get going?” [16, 17]. These questions are the same as proposed by Fried and colleagues. Response options were slightly different: “always, most of the times, sometimes, occasionally, never”, compared to “rarely or none of the time (<1 day), some or a little of the time (1–2 days), a moderate amount of the time (3–4 days), most of the time” in Fried’s version. This criterion was met when participants answered: “always or most of the times” to at least one of the two questions. *Low physical activity* was not measured by using the Minnesota Leisure Time Activity Questionnaire, as proposed by Fried and colleagues. Instead, a slightly adjusted version of the Short Questionnaire to Assess Health-enhancing physical activity (SQUASH) was used [18]. Participants had to answer questions about how many times a week they spent time walking, cycling, gardening, doing odd jobs or exercising/playing sports. For each activity, they had to report how much time they spent engaged in that activity on each occasion. Kilocalories per week were calculated. The results were stratified by gender and compared with the cut-off values as described by Fried and colleagues (men 383 kcal/week, women 270 kcal/week). If a person used fewer kcals per week this criterion was met. *Slowness/walk time* was measured using the question: “Can you reach the other side of the road when the light turns green at a zebra crossing?” We developed this question ourselves. If the participant chose any reply other than “yes, without any trouble”, the criterion was met. *Weakness/grip strength* was measured by asking the question: “Do you experience difficulties in daily life because of low grip strength?” This question was derived from the Tilburg Frailty Indicator [8]. If the participant answered “yes”, the criterion was met.

The stages of frailty, based on the Fried criteria, were defined as follows: a score of 0 means that a person is robust or not frail. Persons with a score of 1 or 2 are at intermediate risk for adverse outcomes or are considered to be pre-frail. A score of 3–5 indicates that someone is frail [5].

#### Perceived health and healthcare use

One question was asked regarding perceived health: “How well is your health in general?” The question could be answered on a 5-point Likert scale with answer choices ranging from “very good” to “very poor”. The use of healthcare services was measured by reporting any contact with a general practitioner within the last two months. The participants also had to provide details regarding the healthcare professional they had contacted over the past twelve months. The healthcare providers were already specified: medical specialist, dietician, occupational therapist, physiotherapist, homecare (nursing care and household care) and social worker.

#### Social domain

Wenger and colleagues developed an 8-item questionnaire regarding social network [19]. The scores divided people into five types of support networks: family dependent, locally integrated, local self-contained, wider community focused, and private restricted. The family dependent and private restricted support networks are characterised by a limited number of people that could provide support. The locally integrated and wider community-focused support networks are larger networks. Wenger and colleagues found that these network types were consistent with the availability of informal support and the use of healthcare services [19]. In addition, one question was asked about the use of informal care over the past 12 months. Loneliness was measured by using the De Jong-Gierveld Loneliness Scale [20]. This is an 11-item scale, with questions such as “I miss having a really close friend”, which allows the participants to choose from three answer choices: “yes”, “more or less” or “no”. A higher score indicates more feelings of loneliness.

#### Psychological domain

The 10-item Kessler Psychological Distress Scale (K-10) was used to measure psychological distress [21]. This questionnaire is comprised of questions such as: “During the last four weeks, about how often did you feel depressed?” The five-category response scale ranged from “all of the time” (score 5) to “never” (score 1). A higher total score indicated higher levels of psychological distress. Mastery was assessed by using Pearlin and Schooler’s instrument [22]. Seven statements, such as: “I have little control over the things that happen to me”, are answered using a 5-point scale, ranging from “I totally agree” to “I totally disagree”. The higher the total score, the more the respondent thinks that life-chances are under one’s own control. Self-management was measured using the short version of the Self-Management Ability Scale (SMAS-S) [23]. The SMAS-S consists of six three-item subscales (taking initiative, investment behaviour, variety, multifunctionality, self-efficacy and positive frame of mind), which reflect core abilities to form the construct of self-management of well-being [24]. Response options were slightly adjusted so that every question had six possible answers. Therefore, the final scores range from 1 to 6, with a higher score indicating more self-management abilities.

#### Physical domain

Chronic diseases were measured by asking participants whether or not they suffered from one or more of the following chronic diseases: diabetes, stroke/cerebral haemorrhage/cerebral infarction, myocardial infarction, other cardiac diseases, cancer, asthma, chronic obstructive pulmonary disease (COPD), hip or knee arthrosis, chronic joint inflammation, or back problems (incl. hernia). For cancer and myocardial infarction the participants had to report if they ever had the diseases. For all of the other diseases, they had to report whether they suffered from the disease over the past twelve months.

IADL-disability (Instrumental activities of daily living) was measured using a seven-item subscale from the Groningen Activity Restriction Scale (GARS) [25, 26]. The subscale is comprised of questions, such as “Can you fully independently prepare dinner?” The items were answered on a four-point scale, with answers ranging from “Yes, without any difficulty” to “No, only with someone’s help”. Scores range from 7 to 28 points, with a higher score indicating a higher level of IADL-disability. Physical limitations were assessed using the Organization for Economic Cooperation and Development (OECD) long-term disability questionnaire [27]. In this study, we used a six-item version, as used by the Community Health Service. This version is comprised of questions about problems with hearing, vision, bending, and walking 400 metres. The number of items that people indicated as problematic were used for analysis.

### Statistical analysis

The central focus of this study was to describe the levels of functioning across various domains. Descriptive statistics were used to present demographic characteristics of the study population and the levels of functioning. Associations between scores in the three health domains and the frailty stages were analysed using Kendall’s tau for nominal and ordinal variables, and analyses of variance (ANOVA) for all other variables (P < 0.05).These associations were also studied separately for men and women, as older women are more likely to be frail. Where available, missing data for all of the included instruments were handled as proposed by the original authors. Fried and colleagues excluded people with three or more missing frailty components. Missing data with respect to the Fried criteria in our study were handled more strictly than originally proposed by the authors. To reduce the number of misclassifications, only one missing value was allowed when a person had a valid Fried score of 0–2. If a person had a valid Fried score of 3 points or more, two missing values were allowed, because this would not cause misclassification. The analyses were performed using IBM SPSS Statistics software version 19.

## Results

The characteristics of the study population are displayed in Table 1. The 8,684 participants were 65 to 98 years of age (mean age 74.2 ± 6.4 yrs.), with slightly more men (53.2 %) than women. The majority of the participants (68.8 %) was married or living together, and more than half of the population had a lower level of education. Nearly 60 % rated their health as very good or good. Almost 51 % had visited their general practitioner during the previous two months, and nearly two-thirds visited a medical specialist over the previous twelve months.

**Table 1:** Demographic characteristics, perceived health and healthcare use according to the three frailty stages

		All	Non-frail	Pre-frail	Frail	*P*-value
		n = 8684	n = 5488 (63.2 %)	n = 2441 (28.1 %)	n = 755 (8.7 %)	
Age (yrs.) (mean ± SD)		74.2 (±6.4)	72.9 (±5.9)	75.9 (±6.5)	78.2 (±6.8)	<0.001
Age groups	65-74	4510 (51.9 %)	3314 (60.4 %)	985 (40.4 %)	211 (27.9 %)	<0.001
	75-84	3597 (41.4 %)	1979 (36.1 %)	1215 (49.8 %)	403 (53.4 %)	
	85+	577 (6.6 %)	195 (3.6 %)	241 (9.9 %)	141 (18.7 %)	
Gender (male)		4619 (53.2 %)	3366 (61.3 %)	999 (40.9 %)	254 (33.6 %)	<0.001
Marital status	married/living together	5837 (68.8 %)	4056 (75.2 %)	1422 (60.2 %)	359 (48.9 %)	<0.001
	unmarried/divorced	721 (8.5 %)	392 (7.3 %)	231 (9.8 %)	98 (13.4 %)	
	widowed	1931 (22.7 %)	943 (17.5 %)	711 (30.1 %)	277 (37.7 %)	
Level of education	low	4903 (58.6 %)	2796 (52.2 %)	1554 (67.4 %)	553 (78.8 %)	<0.001
	medium	1783 (21.3 %)	1254 (23.4 %)	415 (18.0 %)	114 (16.2 %)	
	high	1681 (20.1 %)	1309 (24.4 %)	337 (14.6 %)	35 (5.0 %)	
*Perceived health and healthcare use*						
Perceived health (%)	very good	7.3	10.5	2.2	0.1	<0.001
	good	50.9	64.4	33.8	6.1	
	fair	35.6	24.4	55.5	53.9	
	poor	5.6	0.7	8.1	34.1	
	very poor	0.6	0.1	0.4	5.8	
Healthcare use (past 12 months) (%)	general practitioner <2 months	50.9	43.3	61.3	74.3	<0.001
	medical specialist	64.2	57.3	73.9	83.5	<0.001
	dietician	9.6	6.9	13.1	19.3	<0.001
	occupational therapist	4.1	1.7	6.2	16.8	<0.001
	physiotherapist	35.7	29.2	45.4	53.0	<0.001
	homecare (nursing care and household care)	19.1	6.4	31.9	68.1	<0.001
	social work	2.4	1.0	3.6	9.4	<0.001

**Fig. 1:**
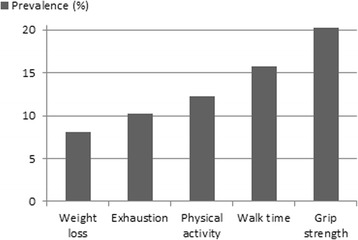
Prevalence of each frailty criterion as proposed by Fried and colleagues

Figure 1 shows the prevalence of each frailty criterion. In this study, 20 % of the participants reported problems with grip strength. Weight loss was reported less often than were the other problems (8 %). The total number of frailty components that were present in the study population is shown in Table 2. In total, 63.2 % of the participants were not frail, 28.1 % were pre-frail and 8.7 % were frail. There were differences between men and women. Men were more often not frail (72.9 % vs. 52.2 %) whereas women were more often pre-frail (35.5 % vs. 21.6 %) or frail (12.3 % vs. 5.5 %).

**Table 2:** Distribution of frailty sum scores

Frailty components	Total	Men	Women
	n = 8684	n = 4619	n = 4065
0	5488 (63.2 %)	3366 (72.9 %)	2122 (52.2 %)
1	1691 (19.5 %)	705 (15.3 %)	986 (24.3 %)
2	750 (8.6 %)	294 (6.4 %)	456 (11.2 %)
3	468 (5.4 %)	154 (3.3 %)	314 (7.7 %)
4	244 (2.8 %)	88 (1.9 %)	156 (3.8 %)
5	43 (0.5 %)	12 (0.3 %)	31 (0.8 %)

When comparing the three different stages, frail people appeared to be older, were more likely to be female, were more often unmarried or living alone, and had a lower level of education compared to their pre-frail and non-frail counterparts (Table 1). Pre-frail participants had intermediate scores between the scores of the frail and non-frail participants. Perceived health was worse when someone was frailer, and frail older people used more health care services. All of the aforementioned differences were statistically significant.

Table 3 shows the results of the measurements for the various health domains. Data are presented for the total study population, as well as per frailty stage. The scores of the non-frail people were more favourable than were those of the pre-frail people, and the scores of the pre-frail people were more favourable than were the ones of the frail people.

**Table 3:** Scores for the total population in the social, psychological and physical domains according to the three frailty stages

		All	Non-frail	Pre-frail	Frail	*P*-value
		n = 8,684	n = 5,488	n = 2,441	n = 755	
*Social domain*						
Social network type (%)	family dependent	22.5	21.8	22.7	27.8	0.049
	locally integrated	40.1	42.5	37.0	31.5	
	local self-contained	21.6	20.6	24.0	21.8	
	wider community focused	8.2	9.2	7.4	3.7	
	private restricted	7.6	6.0	8.9	15.2	
Informal care use in past 12 months (%)	yes, and still present	14.3	3.9	23.8	60.6	<0.001
	no	84.3	95.1	74.2	37.7	
Loneliness (De Jong-Gierveld, 0–11)^a^		3.0 (±3.3)	2.3 (±2.8)	3.8 (±3.5)	5.3 (±3.7)	<0.001
*Psychological domain*						
Psychological distress (K-10, 10–50)		15.9 (±6.4)	13.6 (±4.0)	18.4 (±6.6)	25.3 (±8.2)	<0.001
Mastery (Pearlin & Schooler, 7–35)		25.8 (±5.6)	27.3 (±4.8)	24.1 (±5.6)	19.2 (±5.6)	<0.001
Self-management (SMAS-S, 1–6)		4.1 (±0.7)	4.2 (±0.6)	3.9 (±0.7)	3.4 (±0.8)	<0.001
*Physical domain*						
Chronic diseases (%)	diabetes	16.8	12.5	22.0	31.8	<0.001
	stroke/cerebral haemorrhage or infarction (past 12 months)	1.7	0.9	2.5	4.9	<0.001
	myocardial infarction (ever)	12.6	9.8	15.2	25.0	<0.001
	other heart conditions (CHF or AP) (past 12 months)	8.0	4.6	12.4	19.2	<0.001
	cancer (ever)	18.8	16.8	21.3	25.9	<0.001
	asthma or COPD (past 12 months)	15.5	11.1	21.0	28.5	<0.001
	hip or knee arthrosis (past 12 months)	41.2	30.2	56.2	69.1	<0.001
	chronic joint inflammation (past 12 months)	15.2	7.3	24.8	40.0	<0.001
	back problems (incl. hernia) (past 12 months)	21.3	14.1	29.3	44.7	<0.001
Number of chronic diseases (%)	0	29.0	38.3	15.6	5.0	<0.001
	1	32.1	36.0	27.6	18.3	
	2	19.8	16.9	25.4	22.8	
	≥3	19.1	8.8	31.4	53.9	
GARS IADL-disability (7–28)		11.2 (±5.2)	9.2 (±3.3)	13.0 (±5.4)	19.7 (±5.4)	<0.001
OECD disability	1 or more disabilities (%)	29.4	12.0	49.0	93.9	<0.001

^a^Preferable score is underlined

In the social domain, frail older people more often had a family dependent or a private restricted support network. These network types were characterised by a limited number of people that could offer support. The locally integrated and wider community-focused support networks (both large support networks) were more often present in non-frail older people. A larger proportion of the frail older people used informal care (60.6 %) during the past 12 months compared to the pre-frail (23.8 %) and non-frail older people (3.9 %). They also reported more feelings of loneliness.

Measurements in the psychological domain showed that frail older people experienced more psychological distress (K-10 score 25.3 ± 8.2) than did their pre-frail (18.4 ± 6.6) and non-frail (13.6 ± 4.0) counterparts. They also had a lower sense of mastery and had less self-management abilities.

Measurements in the physical domain illustrated that all chronic diseases were more present among the frail older participants. More than half of the frail older people suffered from three or more chronic diseases. Scores on the GARS indicated that frail older people also had more problems with IADL activities. On the OECD disability questionnaire, 93.9 % of the frail participants reported at least one disability, compared to 49.0 % of the pre-frail and 12.0 % of the non-frail older people. Analyses showed that the differences for all of the scores across the domains were statistically significant between all three frailty stages.

In addition, the associations, as described in Table 3, were studied separately for men and women (Tables 4 and 5 respectively). All of the results showed the same statistically significant differences that were reported for the total study population, except for social network type (men: P = 0.211, women: P = 0.111).

**Table 4:** Scores for men in the social, psychological and physical domains according to the three frailty stages

		All	Non-frail	Pre-frail	Frail	*P*-value
		n = 4,619	n = 3,366	n = 999	n = 254	
*Social domain*						
Social network type (%)	family dependent	22.0	21.1	23.3	28.4	0.211
	locally integrated	41.1	43.5	36.0	28.9	
	local self-contained	21.1	19.9	24.4	23.7	
	wider community focused	8.1	8.9	6.2	3.9	
	private restricted	7.8	6.6	10.1	15.1	
Informal care use in past 12 months (%)	yes, and still present	10.6	3.2	23.6	59.4	<0.001
	no	88.7	96.2	75.6	40.2	
Loneliness (De Jong-Gierveld, 0–11)^a^		2.8 (±3.1)	2.3 (±2.8)	3.9 (±3.5)	5.1 (±3.7)	<0.001
*Psychological domain*						
Psychological distress (K-10, 10–50)		15.0 (±6.0)	13.2 (±3.8)	18.5 (±7.0)	25.3 (±8.4)	<0.001
Mastery (Pearlin & Schooler, 7–35)		26.4 (±5.5)	27.6 (±4.7)	23.9 (±5.8)	18.6 (±5.4)	<0.001
Self-management (SMAS-S, 1–6)		4.1 (±0.7)	4.2 (±0.6)	3.8 (±0.7)	3.3 (±0.8)	<0.001
*Physical domain*						
Chronic diseases (%)	diabetes	17.0	13.8	24.6	30.6	<0.001
	stroke/cerebral haemorrhage or infarction (past 12 months)	1.8	1.0	3.3	7.5	<0.001
	myocardial infarction (ever)	17.1	13.7	23.9	36.4	<0.001
	other heart conditions (CHF or AP) (past 12 months)	8.0	5.1	14.4	21.5	<0.001
	cancer (ever)	19.0	17.1	22.9	29.0	<0.001
	asthma or COPD (past 12 months)	15.2	11.0	24.5	32.4	<0.001
	hip or knee arthrosis (past 12 months)	31.7	25.8	44.8	56.2	<0.001
	chronic joint inflammation (past 12 months)	10.9	7.0	18.8	32.2	<0.001
	back problems (incl. hernia) (past 12 months)	18.8	14.3	28.4	39.8	<0.001
Number of chronic diseases (%)	0	31.0	38.0	14.0	5.5	<0.001
	1	33.2	35.2	29.6	21.7	
	2	18.8	17.2	23.4	21.7	
	≥3	17.0	9.6	33.0	51.2	
GARS IADL-disability (7–28)		11.4 (±5.2)	9.9 (±3.7)	14.3 (±5.8)	20.8 (±5.6)	<0.001
OECD disability	1 or more disabilities (%)	23.8	11.7	47.6	92.4	<0.001

^a^Preferable score is underlined

**Table 5:** Scores for women in the social, psychological and physical domains according to the three frailty stages

		All	Non-frail	Pre-frail	Frail	*P*-value
		n = 4,065	n = 2,122	n = 1,442	n = 501	
*Social domain*						
Social network type (%)	family dependent	23.2	22.9	22.2	27.5	0.111
	locally integrated	38.8	41.0	37.7	32.9	
	local self-contained	22.3	21.6	23.7	20.8	
	wider community focused	8.4	9.6	8.3	3.6	
	private restricted	7.3	5.0	8.0	15.2	
Informal care use in past 12 months (%)	yes, and still present	18.7	5.1	23.9	61.2	<0.001
	no	79.2	93.2	73.2	36.5	
Loneliness (De Jong-Gierveld, 0–11)^a^		3.2 (±3.4)	2.3 (±2.9)	3.8 (±3.6)	5.5 (±3.8)	<0.001
*Psychological domain*						
Psychological distress (K-10, 10–50)		17.0 (±6.7)	14.2 (±4.3)	18.3 (±6.2)	25.4 (±8.1)	<0.001
Mastery (Pearlin & Schooler, 7–35)		25.0 (±5.7)	26.9 (±4.8)	24.2 (±5.5)	19.5 (±5.7)	<0.001
Self-management (SMAS-S, 1–6)		4.1 (±0.7)	4.3 (±0.6)	4.0 (±0.7)	3.5 (±0.8)	<0.001
*Physical domain*						
Chronic diseases (%)	diabetes	16.5	10.4	20.2	32.5	<0.001
	stroke/cerebral haemorrhage or infarction (past 12 months)	1.6	0.8	2.0	3.5	<0.001
	myocardial infarction (ever)	7.3	3.5	8.9	19.1	<0.001
	other heart conditions (CHF or AP) (past 12 months)	8.1	3.9	11.0	18.1	<0.001
	cancer (ever)	18.7	16.4	20.1	24.4	<0.001
	asthma or COPD (past 12 months)	15.9	11.3	18.6	26.6	<0.001
	hip or knee arthrosis (past 12 months)	52.0	37.3	64.0	75.5	<0.001
	chronic joint inflammation (past 12 months)	20.1	7.8	29.0	44.0	<0.001
	back problems (incl. hernia) (past 12 months)	24.0	13.9	30.0	47.1	<0.001
Number of chronic diseases (%)	0	26.7	38.6	16.7	4.8	<0.001
	1	30.9	37.4	26.2	16.6	
	2	21.0	16.5	26.8	23.4	
	≥3	21.5	7.5	30.3	55.3	
GARS IADL-disability (7–28)		10.9 (±5.2)	8.1 (±2.2)	12.1 (±4.9)	19.1 (±5.2)	<0.001
OECD disability	1 or more disabilities (%)	35.8	12.4	50.0	94.6	<0.001

^a^Preferable score is underlined

## Discussion

The aim of this study was to describe the levels of physical, psychological and social functioning according to the three Fried frailty stages using a large cohort of Dutch community-dwelling older people. The results demonstrated consistent differences across all three domains between the non-frail, pre-frail and frail older people. Frail people had poorer scores than did their pre-frail and non-frail counterparts , and older people that were pre-frail had intermediate scores that fell between the scores of frail and non-frail older people.

### Social domain

A family dependent or a private restricted support network was more present among the frail older participants. People in the first network type focused on close family relationships and having few good friends. People in the second network type hardly had any contact with family members (except for sometimes a spouse), minimal contact with neighbours or friends, and a lack of wider community contacts or involvement [19]. This makes people in these network types more vulnerable compared to the ones in a locally integrated or wider community focused network. The latter networks result in having more people to depend on in case of need, and people in these larger networks are considered to be more robust [28]. In addition, the non-frail older people in our study more often had a locally integrated or wider community focused network.

When these analyses were conducted stratified for gender, social network type appeared to be the only characteristic that was not statistically significant different between the frailty subgroups, both in men and in women. There does seem to be a trend in the total and the gender subgroup populations, in which there is a shift from broader to smaller network types when people are more frail. The fact that this difference is not statistically significant is probably due to the local self-contained network type, which appears to be rather stable between the frailty stages. However, because of the cross-sectional nature of the data it is unclear what can be considered cause and effect regarding the changes mentioned previously. One can image that reduced physical abilities (i.e. a higher frailty state) may cause people to stay at home more often, leading to a more restricted network. On the other hand, it cannot be excluded that a decrease in social support leads to a decrease in physical abilities.

Frail older people reported higher levels of loneliness compared to non-frail older people. These results were similar to Ní Mhaoláin and colleagues’ findings [29].

#### Psychological domain

Cramm and colleagues showed that people in poor health were more often frail and had less self-management abilities than their counterparts in good health (SMAS-S mean score 3.5 vs. 4.1) [30]. These results were in line with our study: non-frail participants scored 4.2, pre-frail ones 3.9, and frail persons scored 3.4. Andrew and colleagues also found an association between frailty and psychological well-being [31]. However, this was also a cross-sectional study and the direction of the association is, as in our study, uncertain. We agree with their interpretation that it is most likely that there is a bi-directional relationship: a decline in physical functioning (i.e. increasing frailty) may cause a decline in psychological well-being and vice versa.

#### Physical domain

Scores on the measurements in the physical domain were most favourable for the non-frail older people, and worst for the frail people. Because the phenotype of frailty consists of physical measures, we expected the other measurements in the physical domain to show similar characteristics. Pre-frail participants in our study showed an increased number of chronic diseases compared to their non-frail counterparts. The number of chronic diseases was even higher among the frail people. These results were comparable to those in Fried and colleagues’ study [5]. In Fried’s study, cancer was the only chronic condition that was not significantly different between the frailty stages. In our study, there were differences between stages. This supports Fried’s suggestion that non-significance was due to the exclusion of patients under active treatment for cancer [5]. The more frail the participant was, the higher the GARS-score was, indicating more IADL-disability. Cawthon and colleagues also showed that a larger proportion of frail people had at least one IADL limitation compared to pre-frail or non-frail ones [14].

#### Prevalence of frailty criteria

The prevalence estimates of the frailty criteria ‘weight loss’ and ‘grip strength’ in our study were about the same as those found by Fried and colleagues (8 vs. 6 % and 20 vs. 20 % respectively) [5]. The prevalence of other components was lower in our study. Drey and colleagues compared four large epidemiologic studies where the Fried criteria were applied [32]. They showed that the percentages of frail, pre-frail and non-frail people differ between studies. Our study has the highest prevalence of non-frail, the lowest prevalence of pre-frail and a moderate level of frail older people, compared to other studies among community dwelling older people [5, 12–14, 33]. This variation between studies was probably due to several reasons. First, different measurements were used to determine each criterion. For example, ‘exhaustion’ was measured with different questions in the studies, with the prevalence varying from 8 % to 30 %. Furthermore, ‘hand grip strength’ should have ideally been determined by a physical test. In our study, a self-reported question was used instead. Nevertheless, the prevalence in our study was the same as Fried and colleagues [5]. This could be chance or it might be possible to measure this criterion correctly by using our question instead of a physical test [34]. Slowness/walk time was also not measured by a physical test. The question we used is probably not a perfect measure of walking speed, because there is some variability in the speed of the lights turning red/green at a zebra crossing. In other studies self-report questions on walking capability are often used, for example in the study of Woods and colleagues [35]. They used the Rand-36 physical function scale which does not include specific questions on walking speed. More research is therefore needed on optimal self-report questions to replace the physical measurement of slowness/walk time. Overall, the validity of our Fried operationalizations is supported by the dose response association between our Fried scores and those on the functional status scores (GARS IADL and OECD disability, see Table 3).

Second, an important factor involved differences in the inclusion of persons in the study population. Fried and colleagues, for example, excluded patients under active treatment for cancer. Also, more men than women were present in our study, where usually it is vice versa. The study sample was randomly selected, pre-stratified for age and socioeconomic status. As we have no indications for specific reasons for this ‘imbalance’, we think the overrepresentation of men is a coincidence. Third, we handled missing values for the Fried criteria more strictly than did the original authors.

#### Strengths and limitations

The strength of the present study is that we included a large cohort from the general population. Many questions were asked across various domains and all questions were answered at the same time. Throughout the three health domains, a clear and consistent trend was found, indicating more preferable scores for the non-frail population compared to the frail older population. The pre-frail older people had an intermediate score that was between the scores of the other two populations. This trend remained clear when stratifying for gender (except for social network). Although statistical corrections are often made for gender, age and other factors that vary between different stages of frailty, one can argue whether that is necessary from a clinical point of view. Higher levels of physical disfunctioning are associated with higher levels of social and psychological disfunctioning. In practice, impairments in physical functioning can be used by healthcare professionals to detect impairments in other domains. For that aim no adjustments are needed and therefore we did not use multivariate analyses in our study.

Our study had some limitations as well. First, the overrepresentation of people living in a neighbourhood with a low socioeconomic status and people aged 75 years and over may cause differences in frailty prevalence estimates when our results are compared with those in other studies. Nevertheless, it does not influence comparisons between the frailty stages, which was the main focus of this study. Second, the response rate was 54 %. There is no information available on the characteristics of the non-responders. Therefore, it is not fully clear to what extend the results from our sample can be generalised to other community-dwelling populations. Suijker and colleagues' [36] and Van Dalen and colleagues’ [37] studies investigated the differences between respondents and non-respondents in a population of community-dwelling older people (≥70 years). They found that non-respondents more often had ADL dependency, cognitive impairment, a lower socioeconomic status and received more home visits from their general practitioner [36, 37]. Third, as previously stated, the frailty criteria were not all measured exactly as proposed by Fried and colleagues [5]. Another limitation of this study is the cross-sectional design, as it hampers to determine the direction of the associations that were found. Increasing physical frailty could lead to problems in all described domains. However, problems in these other domains might be factors that cause people to become physical frail. For that reason, all results should be interpreted with caution. Longitudinal studies are necessary to gain more insight in the direction of the associations.

#### Implications

All of the results across all domains showed the same trend, indicating more preferable scores for non-frail compared to frail older people, with intermediate scores for the pre-frail people.

The five Fried frailty criteria could help healthcare professionals efficiently identify and treat frail older people, and providing indications for problems in other domains. So, for (first step) screening purposes one might restrict the screening to the five physical Fried criteria and not use a more elaborated (multidimensional) tool. In subsequent assessment of problems and risks one needs a more multidimensional approach, as our data show that often problems in various health domains co-exist. Further longitudinal research is needed to obtain a better view of which factors predict the negative consequences of frailty.

## Conclusions

When older people are categorised according to the three frailty stages, as described by Fried and colleagues, differences in the level of social, psychological and physical functioning can be found between the non-frail, pre-frail and frail persons. Non-frail participants had consistently more preferable scores compared to frail participants, and pre-frail participants had intermediate scores.

## Abbreviations

ANOVA: Analyses of variance
CES-D: Center for epidemiologic studies depression scale
COPD: Chronic obstructive pulmonary disease
GARS: Groningen activity restriction scale
(I)ADL: (Instrumental) Activities of daily living
K-10: 10-item Kessler psychological distress scale
Kcal: Kilocalorie
OECD: Organization for economic cooperation and development
SHARE: Survey of health, aging and retirement in Europe
SMAS-S: Self-management ability scale – short version
SQUASH: Short questionnaire to assess health-enhancing physical activity

## References

[1] Sternberg SA, Wershof Schwartz A, Karunananthan S, Bergman H, Mark Clarfield A (2011). The identification of frailty: a systematic literature review. J Am Geriatr Soc.

[2] Karunananthan S, Wolfson C, Bergman H, Beland F, Hogan DB (2009). A multidisciplinary systematic literature review on frailty: overview of the methodology used by the Canadian Initiative on Frailty and Aging. BMC Med Res Methodol.

[3] Collard RM, Boter H, Schoevers RA, Oude Voshaar RC (2012). Prevalence of frailty in community-dwelling older persons: a systematic review. J Am Geriatr Soc.

[4] Bouillon K, Kivimaki M, Hamer M, Sabia S, Fransson EI, Singh-Manoux A (2013). Measures of frailty in population-based studies: an overview. BMC Geriatr.

[5] Fried LP, Tangen CM, Walston J, Newman AB, Hirsch C, Gottdiener J (2001). Frailty in older adults: evidence for a phenotype. J Gerontol A Biol Sci Med Sci.

[6] Rockwood K, Mitnitski A (2007). Frailty in relation to the accumulation of deficits. J Gerontol A Biol Sci Med Sci.

[7] Mitnitski AB, Mogilner AJ, Rockwood K (2001). Accumulation of deficits as a proxy measure of aging. ScientificWorldJournal.

[8] Gobbens RJ, van Assen MA, Luijkx KG, Wijnen-Sponselee MT, Schols JM (2010). The Tilburg Frailty Indicator: psychometric properties. J Am Med Dir Assoc.

[9] Schuurmans H, Steverink N, Lindenberg S, Frieswijk N, Slaets JP (2004). Old or frail: what tells us more?. J Gerontol A Biol Sci Med Sci.

[10] Barreto Pde S, Greig C, Ferrandez AM (2012). Detecting and categorizing frailty status in older adults using a self-report screening instrument. Arch Gerontol Geriatr.

[11] Macklai NS, Spagnoli J, Junod J, Santos-Eggimann B (2013). Prospective association of the SHARE-operationalized frailty phenotype with adverse health outcomes: evidence from 60+ community-dwelling Europeans living in 11 countries. BMC Geriatr.

[12] Bandeen-Roche K, Xue QL, Ferrucci L, Walston J, Guralnik JM, Chaves P (2006). Phenotype of frailty: characterization in the women’s health and aging studies. J Gerontol A Biol Sci Med Sci.

[13] Ble A, Cherubini A, Volpato S, Bartali B, Walston JD, Windham BG (2006). Lower plasma vitamin E levels are associated with the frailty syndrome: the InCHIANTI study. J Gerontol A Biol Sci Med Sci.

[14] Cawthon PM, Marshall LM, Michael Y, Dam TT, Ensrud KE, Barrett-Connor E (2007). Frailty in older men: prevalence, progression, and relationship with mortality. J Am Geriatr Soc.

[15] Terstegge C, Houben T, Schefman S, Spee H, Hajema K, Mujakovic S, Quadvlieg M, Verberne N: Onderzoeksprotocol Limburgse monitor volwassenen en ouderen. GGD Limburg, 2012.

[16] Schroevers MJ, Sanderman R, van Sonderen E, Ranchor AV (2000). The evaluation of the Center for Epidemiologic Studies Depression (CES-D) scale: Depressed and Positive Affect in cancer patients and healthy reference subjects. Qual Life Res.

[17] Bouma J, Ranchor AV, Sanderman R, Sonderen van E: Het meten van symptomen van depressie met de CES-D: een handleiding. Tweede herziene druk. UMCG/ Rijksuniversiteit Groningen, Research Institute SHARE 2012.

[18] Wendel-Vos GC, Schuit AJ, Saris WH, Kromhout D (2003). Reproducibility and relative validity of the short questionnaire to assess health-enhancing physical activity. J Clin Epidemiol.

[19] Wenger GC (1991). A network typology: From Theory to Practice. J Aging Stud.

[20] de Jong-Gierveld J, Kamphuls F (1985). The Development of a Rasch-Type Loneliness Scale. Appl Psych Meas.

[21] Kessler RC, Andrews G, Colpe LJ, Hiripi E, Mroczek DK, Normand SL (2002). Short screening scales to monitor population prevalences and trends in non-specific psychological distress. Psychol Med.

[22] Pearlin LI, Schooler C (1978). The structure of coping. J Health Soc Behav.

[23] Cramm JM, Strating MM, de Vreede PL, Steverink N, Nieboer AP (2012). Validation of the self-management ability scale (SMAS) and development and validation of a shorter scale (SMAS-S) among older patients shortly after hospitalisation. Health Qual Life Outcomes.

[24] Steverink N, Lindenberg S, Slaets JJ (2005). How to understand and improve older people’s self-management of wellbeing. Eur J Ageing.

[25] Kempen GI, Doeglas DM, Suurmeijer TPBM: Groningen Activity Restriction Scale (GARS): een handleiding. Tweede herziene druk. UMCG/Rijksuniversiteit Groningen, Research Institute SHARE 2012.

[26] Kempen GI, Miedema I, Ormel J, Molenaar W (1996). The assessment of disability with the Groningen Activity Restriction Scale, Conceptual framework and psychometric properties. Soc Sci Med.

[27] McWhinnie JR (1981). Disability assessment in population surveys: results of the O.E.C.D. Common Development Effort. Rev Epidemiol Sante Publique.

[28] Walker KN, MacBride A, Vachon ML (1977). Social support networks and the crisis of bereavement. Soc Sci Med.

[29] Ni Mhaolain AM, Fan CW, Romero-Ortuno R, Cogan L, Cunningham C, Kenny RA (2012). Frailty, depression, and anxiety in later life. Int Psychogeriatr.

[30] Cramm JM, Twisk J, Nieboer AP (2014). Self-management abilities and frailty are important for healthy aging among community-dwelling older people; a cross-sectional study. BMC Geriatr.

[31] Andrew MK, Fisk JD, Rockwood K (2012). Psychological well-being in relation to frailty: a frailty identity crisis?. Int Psychogeriatr.

[32] Drey M, Pfeifer K, Sieber CC, Bauer JM (2011). The Fried frailty criteria as inclusion criteria for a randomized controlled trial: personal experience and literature review. Gerontology.

[33] Santos-Eggimann B, Cuenoud P, Spagnoli J, Junod J (2009). Prevalence of frailty in middle-aged and older community-dwelling Europeans living in 10 countries. J Gerontol A Biol Sci Med Sci.

[34] Simard J, Chalifoux M, Fortin V, Archambault MJ, St-Cerny-Gosselin A, Desrosiers J (2012). Could questions on activities of daily living estimate grip strength of older adults living independently in the community?. J Aging Res.

[35] Woods NF, LaCroix AZ, Gray SL, Aragaki A, Cochrane BB, Brunner RL (2005). Frailty: emergence and consequences in women aged 65 and older in the Women’s Health Initiative Observational Study. J Am Geriatr Soc.

[36] Suijker JJ, Buurman BM, van Rijn M, van Dalen MT, Ter Riet G, van Geloven N (2014). A simple validated questionnaire predicted functional decline in community-dwelling older persons: prospective cohort studies. J Clin Epidemiol.

[37] van Dalen MT, Suijker JJ, MacNeil-Vroomen J, van Rijn M, van Charante EP M, de Rooij SE (2014). Self-report of healthcare utilization among community-dwelling older persons: a prospective cohort study. PloS ONE.

